# Evolution of candidate transcriptional regulatory motifs since the human-chimpanzee divergence

**DOI:** 10.1186/gb-2006-7-6-r52

**Published:** 2006-06-29

**Authors:** Ian J Donaldson, Berthold Göttgens

**Affiliations:** 1 Department of Haematology, Cambridge Institute for Medical Research, University of Cambridge, Cambridge, CB2 2XY, UK

## Abstract

**Background:**

Despite the recent completion of the chimpanzee genome project, few functionally significant sequence differences between humans and chimpanzees have thus far been identified. Alteration in transcriptional regulatory mechanisms represents an important platform for evolutionary change, suggesting that a significant proportion of functional human-chimpanzee sequence differences may affect regulatory elements.

**Results:**

To explore this hypothesis, we performed genome-wide identification of conserved candidate transcription-factor binding sites that have evolved since the divergence of humans and chimpanzees. Analysis of candidate transcription-factor binding sites conserved between mouse and chimpanzee yet absent in human indicated that loss of candidate transcription-factor binding sites in the human lineage was not random but instead correlated with the biologic functions of associated genes.

**Conclusion:**

Our data support the notion that changes in transcriptional regulation have contributed to the recent evolution of humans. Moreover, genes associated with mutated candidate transcription-factor binding sites highlight potential pathways underlying human-chimpanzee divergence.

## Abstract 2

The genome-wide identification of conserved candidate transcription-factor binding sites that have evolved since the human-chimpanzee divergence supports the notion that changes in transcriptional regulation have contributed to the recent human evolution.

## Background

Comprehensive analysis of the draft chimpanzee genome confirmed that the human and chimpanzee genomes are 98.8% identical [1]. Given the dramatic behavioral and developmental differences that have arisen since their divergence from a common ancestor 6-7 million years ago [2], the question therefore arises of how these phenotypic differences are reflected at the genome sequence level.

Two major consequences of DNA sequence alteration are changes in protein coding sequence and changes that affect spatiotemporal and/or quantitative aspects of gene expression. The latter includes post-transcriptional aspects of gene expression, such as sequence changes that affect alternative splicing or RNA stability. However, one of the major causes of changed gene expression is likely to be changes in gene regulatory sequences, such as promoters, enhancers, and silencers [3,4]. Such sequence changes might increase or decrease affinities for specific transcription factors, or indeed result in the acquisition of new binding sites.

Comparative gene expression profiling can identify subsets of genes for which expression levels differ between human and chimpanzee tissues [5-8]. This provides a potentially powerful approach to identifying those differences in the genome that are responsible for the different expression patterns or levels. Accordingly, a recent study [8] showed that the degree of sequence divergence in aligned human and chimpanzee core promoters correlated with the divergence of gene expression levels. However, two important issues remain unresolved. First, which specific DNA sequence changes are responsible for the altered levels of gene expression? (These will not be restricted to core promoters and may be located far away in enhancers.) Second, which of the expression changes contribute to the phenotypic differences between humans and chimpanzees?

When compared with lower eukaryotes, the greater biologic complexity of mammalian genomes is thought largely to be a result of intricate mechanisms of gene regulation [9]. Consequently, although deciphering gene regulatory mechanisms is a prerequisite to understanding human genome function, the complexity of regulatory mechanisms raises several problems. The connectivity or 'hard wiring' of transcriptional regulatory networks is achieved through transcription factors binding specific sequence motifs in gene regulatory regions [10]. However, the identification of functional regulatory motifs is hampered by the fact that transcription factor binding sites (TFBSs) are often short (four to six nucleotides) and degenerate. This means they occur by chance alone in the genome, thereby obscuring functional sites. Moreover, and unlike in simpler genomes such as worm or yeast, in mammalian genomes TFBSs are frequently located outside the proximal promoter of a gene in distal 5' and 3' enhancers or within introns.

One method by which discovery of functional sites can be improved is by using phylogenetic footprinting that focuses on the areas of sequence conservation between two or more species [11]. For example, comparison of human and mouse sequences (separated by 70 million years) is a widely used approach to identifying gene regulatory sequences [12-15]. However, this method is not sensitive enough to detect functional differences between evolutionarily 'close' species, such as human and chimpanzee. Phylogenetic shadowing represents a possible alternative because it was designed for the analysis of closely related genomes. However, this method requires sequences from multiple closely related species to function effectively [16].

Here, we conducted a genome-wide comparative analysis of candidate TFBSs that have changed since the human-chimpanzee divergence. We show that, when categorized based on Gene Ontology (GO) annotation [17], changes in candidate regulatory motifs correlate with genes that perform specific biologic functions, principally the sensory perception of chemical stimulus (smell). Our data therefore suggest that positive selection of altered gene regulatory programs played a significant role in human evolution.

## Results

### Identification of candidate regulatory TFBSs that have evolved since human-chimpanzee divergence

Because neither human-mouse comparisons nor phylogenetic shadowing appeared to be useful strategies in identifying candidate regulatory motifs that have evolved since human-chimpanzee divergence, we devised an alternative strategy. Our approach initially entailed the identification of candidate binding sites that are conserved in mouse-human and mouse-chimpanzee whole genome alignments. Data files containing these sites can be downloaded from our website [18]. This approach therefore uses sequence conservation as the criterion to enrich for functional TFBSs. The mouse genome was used as the common reference sequence for both alignments to facilitate the identification of those binding sites conserved between mouse and chimpanzee but not between mouse and human. The TFBS consensus sequences chosen for the above searches were the top 30 motifs from a recently published seminal study [19] that identified common regulatory sites conserved in human, dog, mouse, and rat genomes.

**Figure 1:**
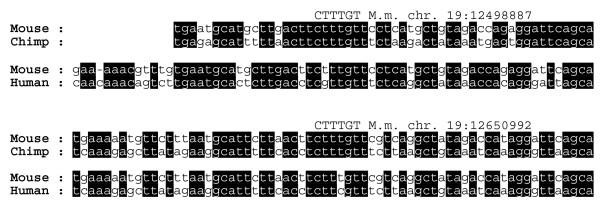
Candidate TFBSs absent in human. Two examples of candidate motifs lost in the human genome. Shown are the genome positions of candidate TFBSs together with extracts from mouse-chimpanzee and mouse-human whole genome alignments. TFBS, transcription factor binding site.

The number of sites conserved in the mouse-chimpanzee and mouse-human genome alignments ranged from fewer than 20 to more than 200,000, depending on the complexity of the TFBS consensus sequence (see supplementary data at our website [18]). Although the numbers of conserved TFBSs were similar for both comparisons, fewer conserved sites were found for mouse-chimpanzee than for mouse-human alignments. This is most likely due to the draft status of the chimpanzee genome, in which some sequence is still missing or may contain errors [1,20]. Using the mouse genome as the common reference sequence allowed us to compare corresponding lists of TFBSs directly and, for example, to identify those sites that were conserved between mouse and chimpanzee but absent in human (referred to as human mutated sites; available from our website [18]). In subsequent analysis we concentrated on the human mutated sites because of the draft status of the chimpanzee genome sequence. For the converse, namely those TFBSs that were conserved in mouse and human but absent in chimpanzee, some chimpanzee motifs might be counted as absent because of missing or erroneous chimpanzee sequence. By contrast, given the high accuracy of current human genome sequence builds, most mouse-chimpanzee motifs not present in human are likely to be results of mutations in the human lineage rather than a result of sequencing artifacts. Two examples of human specific sequence changes in candidate TFBSs are shown in Figure 1. Data files containing binding site positions conserved in mouse and chimpanzee but absent in the human genome can be downloaded from our website [18]. These files therefore represent a genome-wide resource cataloging candidate TFBSs that have evolved in human and chimpanzee lineages since their last common ancestor.

### Genome-wide distribution of human mutated motifs

**Figure 2:**
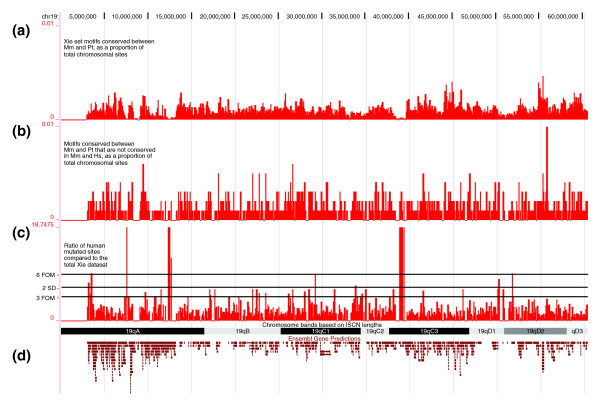
Distribution of candidate TFBSs plotted against mouse chromosome 19 in the UCSC genome browser. **(a) **Distribution of mouse-chimpanzee conserved TFBSs. Bars indicate the number of TFBSs per 50 kb divided by the total number of TFBSs on chromosome 19. **(b) **Distribution of human mutated TFBSs plotted as in panel a. **(c) **Over-representation of human mutated sites relative to the total mouse-chimpanzee TFBS data set. Shown is the ratio b/a of the two plots above. The three horizontal lines indicate sixfold over median threshold, mean plus two standard deviations, and threefold over median threshold (from top to bottom). **(d) **Structure of mouse chromosome 19 with chromosome bands and Ensembl gene predictions taken from the UCSC genome browser. kb, kilobase; TFBS, transcription factor binding site; UCSC, University of California at Santa Cruz.

We then addressed the distribution of candidate TFBSs mutated since the human-chimpanzee divergence. As our searches were based on the top 30 TFBS consensus sequences from the report by Xie and coworkers [19], it was important to control for any potential bias in their genome-wide distribution. We therefore divided each chromosome into 50 kilobase (kb) nonoverlapping tiles and calculated the following: the relative number of mouse-chimpanzee conserved sites as a proportion of these sites on the chromosome; the relative number of human mutated sites as a proportion of these sites on the chromosome; and the ratio of the relative number of human mutated sites over the relative number of mouse-chimpanzee conserved sites (fold enrichment). To facilitate subsequent analysis of those parts of the genome that were highly enriched in human mutated sites, the mean and median values of fold enrichment were calculated for each chromosome. Subsequently, enrichment thresholds were set at mean plus twofold standard deviation as well as threefold and sixfold over median. These three levels equated to 6.54-fold, 5.17-fold, and 10.54-fold enrichment, respectively, averaged over all chromosomes (except X and Y). Chromosome X had significantly higher fold enrichment at 9.68, 6.33, and 12.67, respectively. Chromosome Y only had two enriched tiles and we set enrichment thresholds to 6, 5, and 10, respectively. The chromosome specific data were plotted against the University of California at Santa Cruz (UCSC) mouse genome browser (March 2005/mm6/build 34) using custom tracks for each chromosome. The files are available from our website [18]. Figure 2 is an example of the data plotted against mouse chromosome 19.

The analysis outlined above demonstrated that the TFBSs generated from the consensus sequences derived by Xie and coworkers [19] are not distributed evenly across the genome, thus emphasizing the need to control the distribution of TFBSs lost in human. Importantly, however, the distribution of TFBSs lost in human did not simply shadow the distribution of the total TFBS data set (Figure 2c). This allowed us to characterize further those regions of the genome that were relatively enriched for candidate TFBSs lost in the human genome.

### Analysis of genomic regions enriched in candidate TFBSs mutated since the human-chimpanzee divergence

The candidate regulatory TFBS consensus sequences used in the present study were based on the top 30 hits from a recent study [19] that was designed to identify sequence TFBSs that play an important role in gene regulation. Importantly, no specific regulatory functions have been assigned to these sites as yet. Therefore, detailed analysis of whether particular over-represented human mutated motifs recurrently occur in the vicinity of specific groups of genes is at present limited in its ability to yield deep biological insight, but it may become useful in the future. Nevertheless, a striking observation was that a small number of biologic functions (as indicated by GO annotation) were statistically over-represented in genes within the vicinity of these TFBSs.

To correlate changes in candidate TFBSs with possible biologic functions, the GOToolBox program 'GO-Stats' was run using the genes identified throughout the genome in areas over-represented by human mutated TFBSs. We also included genes with 25 kb either side of the 50 kb tiles, because sites of interest can be close to the boundary of a tile and may control genes not within the original tile coordinates. First, we cataloged all genes present in tiles enriched in human mutated sites (mean plus two standard deviations as well as threefold and sixfold over median). We focused GO analysis on these groups because we reasoned that regions exhibiting enrichment were more likely to contain at least some biologically important sequence changes than those with lower levels of enrichment. (Of course, this does not exclude the possibility that areas with lower enrichment may contain some biologically important sequence changes.) The input files for GOToolBox can be downloaded from our website [18]. Several GO terms were either statistically over-represented (enriched; *P *< 0.01) or under-represented (depleted; *P *< 0.01) relative to the distribution of the same terms in the complete mouse genome (Table 1). Similar results were obtained when we analyzed our data set with an analogous tool made available as part of the Panther Classification System [21] (data not shown).

**Figure 3:**
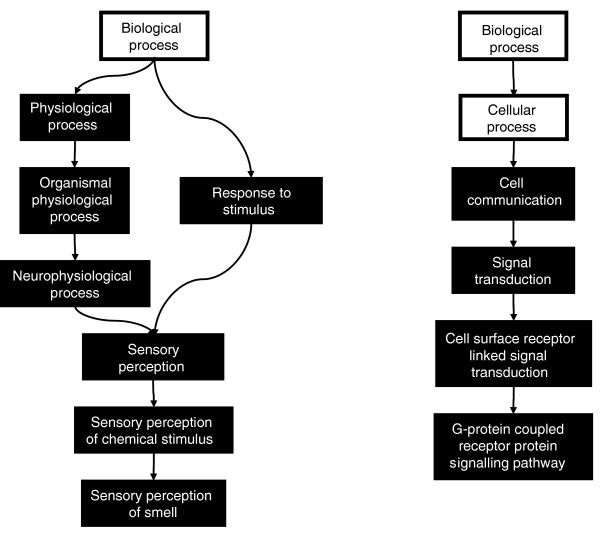
GO trees showing the relationships between 'biological process' terms associated with genes containing TFBSs mutated in human. Filled boxes represent GO terms that were over-represented in genes associated with the conserved TFBSs analyzed in the present study (sixfold over median enriched set). Ontology relationships were confirmed using the GOToolBox ontology browser. The 11 enriched GO terms could be integrated into only two functionally related GO trees. GO, Gene Ontology; TFBS, transcription factor binding site.

Eleven GO terms were significantly over-represented in the most restrictive group of genes (sixfold over median density of human mutated sites). When visualized as a GO tree (a facility of GOToolBox), it became apparent that these GO terms were biologically linked in two clusters, culminating in the GO terms 'sensory perception of smell' and 'G-protein-coupled receptor protein signaling pathway' (Figure 3). The fact that a small number of GO terms were over-represented suggests that candidate TFBSs mutated since the human-chimpanzee divergence are not randomly distributed across the genome. Moreover, olfactory receptor genes encode G-protein-coupled receptors, thus linking the two groups of GO terms shown in Figure 3. GOToolBox analysis of the threefold over median enriched set gave similar results with only one difference; 'physiological process' was no longer identified whereas 'response to pheromone' was (data not shown). GOToolBox analysis of the mean plus two standard deviations enriched set again gave similar results. The same 10 overlapping GO annotations were identified, and 'physiological process' was now replaced with 'keratinization' (data not shown).

We next investigated the GO terms that were under-represented in the gene lists derived from candidate TFBSs lost in humans. This analysis should identify biologic functions associated with gene loci where mutation of candidate binding sites was a relatively rare event in recent human evolution. Only five GO terms were significantly under-represented in the sixfold over median group: 'nucleobase, nucleoside, nucleotide and nucleic acid metabolism', 'development', 'cellular physiological process', 'primary metabolism', and 'cell organization and biogenesis'. For the threefold over median set the first three GO terms were found as well as several additional terms: 'transcription, DNA dependent', 'regulation of cellular metabolism', 'regulation of biological process', 'regulation of physiological process', 'regulation of cellular process', 'cellular defense response', and 'regulation of cellular physiological process'. For mean plus two standard deviations, four GO terms were under-represented: three GO terms were the same as for the sixfold over median set ('nucleobase, nucleoside, nucleotide and nucleic acid metabolism', 'development', 'cellular physiological process') and one additional term was also identified ('organ development'). Consistent with a slow rate of evolutionary change at the respective loci, all depleted GO terms describe very fundamental biologic processes that are likely to be conserved across large evolutionary distances. Taken together, our observation that specific GO terms were identified consistently using three different thresholds suggests that this approach may be used to identify potential pathways underlying human-chimpanzee divergence.

## Discussion

It was proposed more than 30 years ago that gene regulatory mutations account for many of the major biologic differences between humans and chimpanzees [22]. This idea has been reinforced by the recent demonstration of widespread heritability of variation in gene expression levels in humans [23]. Nevertheless, a recent theoretic analysis of human and chimpanzee genome sequences [24] argued that, due to small population sizes in primates, selection may not be effective for regulatory mutations. However, concerted functional analysis of specific genes has identified positive selection of regulatory variants during recent primate evolution. For example, comparative analysis of various primates focusing on the factor VII and prodynorphin gene promoters [25,26] demonstrated selection of sequence variants affecting transcriptional activity, thus supporting the hypothesis that regulatory mutations have been important in human evolution. The apparent discrepancies between the theoretical and experimental studies may at least partly be a consequence of the theoretical study treating all bases equally. By contrast, we aimed to focus our analysis on a small subset of noncoding sequence by incorporating sequence conservation and TFBS content criteria, and the data obtained using these criteria are consistent with regulatory evolution having contributed to recent human evolution.

Our approach to restrict analysis to likely regulatory sites is in many ways analogous to previous studies of human-chimpanzee divergence that studied the evolution of protein coding sequences [27,28] and divided sequence alterations into synonymous and nonsynonymous changes. Nonsynonymous changes are more likely to affect protein function than are nonsynonymous ones. By analogy, sequence alterations in candidate TFBSs are more likely to affect gene regulatory mechanisms than changes in noncoding sequence not thought to be involved in regulatory control. The second key aspect of our methodology was to study likely TFBSs in a comparative way in human, chimpanzee, and mouse to enrich further the likely functionality of candidate sites. Restricting the analysis on candidate TFBSs conserved between mouse and chimpanzee but absent in humans was again similar to a principle employed in a recent comparative analysis of human/chimpanzee/mouse coding sequences [29]. The latter study provided strong evidence for non-neutral evolution of coding sequences and, similar to our study, suggested that positive selection during human evolution was affiliated with a subset of biologic processes.

Clearly, the evolutionary pressures acting on coding versus gene regulatory sequences may be different. Nevertheless, 'sensory perception' - the biologic process most strongly associated with positive selection in human protein evolution [29] - was also shown to be over-represented in the present study, and indeed represented the GO term that integrated most of the other terms found to be over-represented. The data presented here therefore suggest that recent divergence of sensory perception of smell between humans and chimpanzees may have occurred at the gene regulatory level as well as the protein sequence level. Interestingly, when a lower threshold of motif over-representation was applied using the Panther classification tool (threefold over median, as opposed to sixfold over median), several GO terms associated with B-cell immune function were identified in addition to the olfactory pathway (data not shown). Interestingly, the GO term 'B-cell function' had not been associated with recent human evolution when human and chimpanzee coding sequences were compared [29]. This may be the result of different evolutionary pressures acting on coding sequences versus regulatory mechanisms.

Two recent studies attempting to uncover general principles governing the recent evolution of human coding and regulatory sequences reached opposing conclusions. According to the neutralist model of evolution between human and chimpanzee genes, divergence of protein coding sequence and expression are tightly linked [1]. By contrast, the selectionist view argues that there is no, or very little, correlation between coding sequence divergence and expression [30,31]. Our analysis was restricted to potential regulatory motifs lost since human-chimpanzee divergence. We therefore did not address the potential for newly created TFBSs. Moreover, we only analyzed those motifs lost during human evolution. Given the potentially different evolutionary pressures acting on coding and regulatory sequences, we would argue that generalized concepts explaining the parallel evolution of coding sequences and regulatory elements may not be applicable in a genome-wide manner. Our observation that olfactory receptor genes exhibit apparent accelerated evolution at both the gene regulatory and protein levels is consistent with the neutralist model of evolution. However, this parallel accelerated evolution may not apply to genes playing a role in B-cell function. By providing a genome-wide catalog of candidate TFBSs mutated since the human-chimpanzee divergence, the present study will not only allow the characterization of general patterns of evolutionary change but also facilitate analysis of specific gene loci.

## Conclusion

Alteration in transcriptional regulatory mechanisms represents an important platform for evolutionary change. This report suggests that a significant proportion of functional human-chimpanzee sequence differences may affect regulatory elements, thus supporting the notion that changes in transcriptional regulation played an important role in recent human evolution. Moreover, by identifying genes associated with mutated candidate binding sites, the present study highlights potential pathways underlying human-chimpanzee divergence.

## Materials and methods

### Discovery of conserved binding sites in aligned genomes and the determination of sites mutated in the human genome

The genome assemblies used in the mouse-human and mouse-chimpanzee sequence alignments were mouse (mm6), human (hg17), and chimpanzee (panTrog1). The localized alignments for each comparison (specifically the axtNet processed alignments) were downloaded from the Genome Bioinformatics group at the UCSC [32]. Conserved binding sites were located using our PERL program TFBSsearch [33], excluding those located in repetitive sequence identified in the alignments by softmasking.

For 30 TFBS consensus sequences, positions of mouse-chimpanzee conserved binding sites were compared with mouse-human conserved binding sites (using a PERL script); those motifs that could not be found to be conserved in the mouse-human alignments were retained for further study. Of these motifs we removed duplicated sites, those located in annotated exons, and areas affected by genomic structural variation (using PERL scripts). Duplicate sites are present as eight of the motifs contain palindromic sequence and our program searches the entire genome on both strands. We chose to remove sites located in annotated mouse exons (both coding and untranslated), the positions of which were retrieved using the Ensembl API v32 [34]. Genomic structural variations are manifest by deletions, insertion, and inversions between the human and chimpanzee genomes [35]. To this end we only considered motifs in areas of the mouse genome that are present in both human and chimpanzee genome pair-wise alignments.

### Localization of Ensembl genes to binding sites mutated in the human genome

For each of the 50 kb tiles (± 25 kb) over-represented by human mutated TFBSs, we employed a PERL script [33] utilizing the Ensembl API v32 to identify all genes in these regions. Gene symbols (represented by the Ensembl identifier 'db_xref: MarkerSymbol') were extracted from each localized gene file and were processed to ensure all gene symbols are only represented once in the data set.

### Identifying gene function using GO

The web-based tool GOToolBox [36,37] was used to identify statistically over-represented or under-represented GO terms in our gene symbol data sets compared with the distribution of the terms among the annotations of the complete genome. The 'Create Dataset' program was used to make a file compatible with the 'GO-Stats' program. To run 'Create Dataset' *Mus musculus *was selected as the target species and 'biological processes' was chosen for the ontology type. Other options were left as default. The resulting file was used with the 'GO-Stats' program employing the hypergeometric statistical test and correction for multiple tests using the Bonferroni method.

## Figures and Tables

**Table 1:** GO terms over-represented in the gene tiles enriched for human mutated sites (at sixfold over median threshold)

GO ID	GO Level	GO Term	*P*
GO:0007606	6,5	Sensory perception of chemical stimulus	1.21 e^-43^
GO:0007608	7,6	Sensory perception of smell	1.31 e^-41^
GO:0007600	5,4	Sensory perception	5.95 e^-41^
GO:0050877	4	Neurophysiological process	2.00 e^-37^
GO:0007186	6	G-protein-coupled receptor protein signaling pathway	3.48 e^-30^
GO:0050896	3	Response to stimulus	2.87 e^-26^
GO:0050874	3	Organismal physiological process	8.04 e^-26^
GO:0007166	5	Cell surface receptor linked signal transduction	8.05 e^-23^
GO:0007165	4	Signal transduction	6.26 e^-15^
GO:0007154	3	Cell communication	1.01 e^-10^
GO:0007582	2	Physiological process	9.89 e^-04^

GO, Gene Ontology.
